# Drawing with Oblique Coordinates: On a Single Case

**DOI:** 10.3233/BEN-2011-0333

**Published:** 2011-08-29

**Authors:** Dario Grossi, Gabriella Santangelo, Giuseppe Carbone, Flavia Giordano, Valentina Gerarda Angelillo, Luigi Trojano

**Affiliations:** ^1^Neuropsychology Lab.Department of PsychologySecond University of NaplesCasertaItaly; ^2^IDC “Hermitage Capodimonte”NaplesItaly; ^3^IDC “Villa delle Magnolie”CastelmorroneCasertaItaly

**Keywords:** Constructional apraxia, spatial coordinates

## Abstract

We describe a patient with right hemisphere damage affected by mild left visuo-spatial neglect and constructional apraxia. During the rehabilitation, he failed to draw a draught-board using horizontal and vertical trajectories, but he performed it successfully using oblique trajectories. These observations suggested an impairment of vertical/horizontal spatial coordinates system. In copying tasks including figure elements in different orientations he drew more accurately components in oblique orientation, whereas failed to reproduce components in horizontal orientation. The patient performed visuospatial perceptual and perceptual-imaginative tasks successfully. From these findings, it is possible to suggest that the oblique coordinate system of reference operates independently of vertical and horizontal coordinate systems in building a complex figure and that, therefore, cardinal orientation do not constitute a reference norm to define oblique orientation, as previously suggested.

## 1. Introduction

It is a consolidated clinical experience that patients with brain damage experience more difficulties in reproducing figures with oblique elements compared to those with horizontal and vertical elements [1,2]; for instance, in copying geometric figures demented patients often "regularize" stimuli containing oblique lines, e.g. tend to draw squares as diamonds [3]. Such observations support the view that oblique lines are more difficult to draw than horizontal-vertical lines [4]. In particular, it has been suggested that oblique orientation is computed on the basis of vertical and horizontal dimensions, at least in the haptic modality [5].

Some neuropsychological case studies seem to confirm that production of lines in different directions can dissociate. A selective impairment to draw horizontal lines, compared to vertical lines, has been described in a patient with degenerative dementia, who showed the same dissociation in perceptual and representational domains too [6]. More recently, a specific inability to produce oblique lines has been described in a patient with constructional apraxia affected by a right-hemisphere infarct in the territory of the middle cerebral artery [7].

Here we describe a patient with mild left visuo-spatial neglect and severe constructional apraxia who showed relatively spared drawing along oblique dimensions. The patient drew quite accurately oblique elements of complex figures, whereas he tended to omit or displace horizontal and/or vertical components. This pattern seems at odd with available clinical evidence and would suggest that oblique coordinates might be

*Corrisponding author: Gabriella Santangelo, Department of Psychology, Second University of Naples, Via Vivaldi 43, 81100 Caserta, Italy. Tel.: +39 0823274774; E-mail: gabrysantangelo@libero.it.
[1]This work was not supported by grants, equipment, drugs, or any combination of these.
No disclose funding was received for this work from any of the following organizations: National Institutes of Health (NIH); Wellcome Trust; Howard Hughes Medical Institute (HHMI); and other(s).

ISSN 0953-4180/11/$27.50 © 2011 – IOS Press and the authors. All rights reserved

Table 1
Formal neuropsychological assessment

| Tests | Score | Equivalent score* |
|---|---|---|
| MMSE | 28/30 | |
| *Memory* | | |
| WAIS-Digit Span | 5 | 3 |
| Verbal span | 4 | 2 |
| Spatial span | 4 | 2 |
| Babcock story recall test | 9/16 | 2 |
| *Frontal functions* | | |
| Verbal judgment task | 42/60 | 2 |
| Verbal fluency | 10.05 | 1 |
| *Constructional Functions* | | |
| Constructional Apraxia Test | 7 | 0 |
| WAIS-Block Design subtest | 0 | – |
| *Visuospatial functions* | | |
| Line cancellation task (Albert test): | | |
| left-sided omissions | 1/11 | – |
| right-sided omissions | 0/10 | – |
| Star cancellation task: | | |
| left-sided omissions | 4/27 | – |
| right-sided omissions | 0/27 | – |
| Sentence reading task: omissions | 0/6 | – |

*Note: where available equivalent scores refer to Italian age- and education-adjusted norms: 0 means below the normal range; 1 to 3 mean within the normal range, at about the 10th, 25th and 50th centile, respectively.

processed independently from horizontal and vertical dimensions in constructional tasks.

## 2. Case report

G.N. was a 56-year-old right-handed male motor mechanic, who suddenly developed a haemorrhagic stroke in the right fronto-temporo-parietal region. Two months after onset clinical assessment revealed left hemiplegia without visual field defects. On formal neuropsychological evaluation (see Table 1), the patient did not show general cognitive defects, and his performance on tasks tapping short-term and long-term memory, and frontal lobe functions was within age- and education-adjusted normal range. The patient showed a few left-sided omission errors in spatial exploration tasks (line and star cancellation and sentence reading), but could draw from memory and copy a clock face without asymmetries. On constructional tasks G.N. made gross visuospatial distortions. For instance, in the Block Design subtest from Wechsler Adult Intelligence Scale G.N. reproduced the items as non-connected diamonds, i.e. rotated the response blocks and could not align them to each other.

The present study origins from a naive observation. During a rehabilitation session, G.N. was asked to blacken cells in a grid to the aim of producing a draught-board; in this constructional task the examiner did not provide specific instructions or designate any starting point. The patient started darkening cells along horizontal and vertical trajectories, but he failed in completing the task. After having acknowledged his own error, G.N. spontaneously decided to repeat the task using oblique trajectories, and starting from right bottom cell; by this latter procedure G.N. performed successfully (Fig. 1).

This performance triggered a specific neuropsychological assessment aimed to verify whether the patient had a specific, and very unusual, sparing of the oblique dimension in constructional tasks, in presence of a gross impairment along horizontal/vertical dimensions. For this purpose, we devised several constructional tasks in which the patient had to reproduce simple vertical, horizontal and oblique lines and complex figures. Moreover, to explore whether analogous impairments could be observed in the visuoperceptual domain (e.g. [6]), and to verify whether left spatial neglect played any role in determining G.N.'s behaviour, we used the same material in perceptual and perceptual-imaginative matching tasks too.

## 3. Special neuropsychological assessment

### 3.1. Constructional tasks

To verify whether the patient showed a dissociation between drawing horizontal lines (impaired) and oblique/vertical lines (spared), as suggested by the draught-board task described above, we devised several constructional tasks, in which we varied stimuli's complexity and position. In the first two tasks a single stimulus constituted by linear elements had to be reproduced with or without spatial cues; in the third task we used one to four multipart figures of different complexity, presented in different spatial locations; in a last, control, task we assessed copying single lines in horizontal, vertical or oblique orientation.

### 3.2. Copying asterisks

#### 3.2.1. Material and methods

Stimuli consisted in three different symbols, depicting a complete asterisk (composed of one vertical line, one horizontal line and two oblique lines intersecting at their centre), or "incomplete asterisks", made of three intersecting lines (e.g., one horizontal line, one verti-

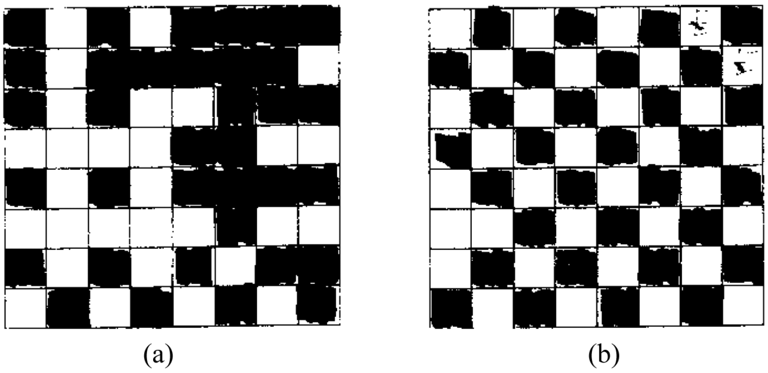

Fig. 1. Draught-boards produced by G.N. following horizontal and vertical trajectories (a) and following oblique trajectories (b).

cal line and one oblique line), or of two intersecting lines (e.g., one horizontal and one vertical line, or two oblique lines). Each stimulus was printed in the upper half of an A4 sheet, and the patient was required to reproduce it in the lower half of the same sheet. A total of 22 stimuli were presented (one at a time).

In scoring responses we adopted a conservative criterion, based on a pilot study on 5 age- and education-matched normal controls: we judged as wrong the responses lacking one or more elements and those in which elements' angular orientation differed for more than 10° (the worst controls' response) with respect to the target.

*3.2.2. Results*

The patient reproduced 12/22 stimuli (55.5%) correctly. Qualitative analysis of the 10 mistakes revealed that they only concerned horizontal segments of figures; for instance, in copying a cross consisting of one oblique and one horizontal arm, G.N. first drew the oblique arm and then was unable to complete the horizontal one in the correct orientation.

### *3.3. Copying asterisks with landmarks*

*3.3.1. Material and methods*

The same stimuli as above were used in a drawing completion task, in which stimuli were printed in the upper half of A4 sheets, whereas in the lower half of the sheet the same stimulus with one element missing was given as a cue for the patient.

Task instructions required the patient to complete the lower figure by drawing the only missing element. A total of 56 drawings were presented in a random order.

Based on a pilot study on age- and education-matched normal controls, we judged as wrong reproductions only lines whose angular orientation differed for more than 13° (the worst controls' response) with respect to the target line.

*3.3.2. Results*

The patient reproduced 32/56 stimuli (57.1%) correctly. All the 24 mistakes consisted in wrong reproductions of horizontal elements (Fig. 2).

### *3.4. Copying complex figures (daisies, windmills and nonsense figures)*

*3.4.1. Material and methods*

Stimuli consisted in complex figures composed by six elements in oblique or horizontal orientations (set 0° as the vertical orientation, elements were set at 45°, 90°, 135°, 225°, 270°, and 315°; see Fig. 3); three types of elements were used (ovals, triangles and nonsense lines) to form daisies, windmills, and non-sense patterns, respectively. One to four stimuli of the same kind were printed on horizontally aligned A4 sheets; together with each stimulus, the patient was presented with a blank A4 sheet placed below the stimulus card, and was required to copy each stimulus on the response sheet, without time constraints. A total of 120 stimuli were randomly presented across separate sessions.

In scoring responses, we considered as correct reproductions those containing all stimuli's elements, without considering their precise orientation; in case of defective reproduction we took into account number and position of omitted or distorted elements.

*3.4.2. Results*

G.N. reproduced all the figures, but he systematically omitted left-sided horizontal elements (74% of omissions), independently of number, spatial position and shape of stimuli. Moreover, he often produced oblique elements first and then laboured to draw the horizontal ones. G.N. was aware of his difficulties in drawing horizontal lines (Fig. 3).

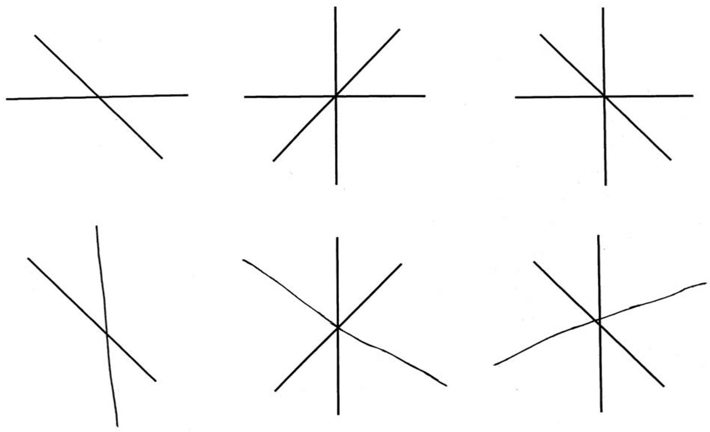

Fig. 2. Copying "incomplete" asterisks with landmark: the patient could not reproduce horizontal elements in the proper orientation (models depicted in the upper row, patient's reproductions in the lower row).

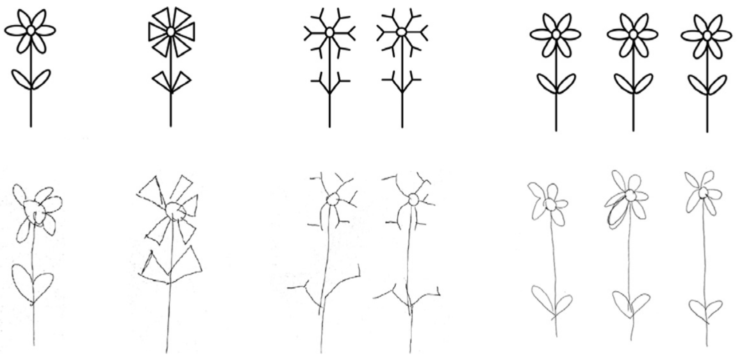

Fig. 3. Copying multipart figures: the patient tended to omit or distort the left-sided horizontal element of each figure, independently from the type, the number or the position of stimuli (models depicted in the upper row, patient's reproductions in the lower row).

### 3.5. Copying simple lines

#### 3.5.1. Material and methods

Simple 10-cm vertical, horizontal or oblique lines were printed on the upper half of A4 sheets, and the patient was required to reproduce each stimulus in the lower half of the sheets. A total of 20 stimuli were presented in random order.

Based on a pilot study on 5 age- and education-matched normal controls, we judged as wrong reproduction only lines whose angular orientation differed for more than 8° (the worst controls' response) with respect to the target line.

#### 3.5.2. Results

G.N. scored 20/20 correct.

### 3.6. Perceptual-imaginative tasks

To evaluate whether errors and omissions in the copying tasks could be ascribed to perceptual deficits or to left spatial neglect, the patient underwent several tasks in which the same stimuli used for the constructional tasks were employed in perceptual and delayed matching tasks.

### 3.7. Perceptual matching of asterisks

#### 3.7.1. Material and methods

We employed the same complete and "uncomplete" asterisks used in the first constructional task. For the purpose of the present experiment two stimuli were printed on an A4 sheet one above the other: in half trials they were identical, whereas in half trials they differed for one element. The patient was required to judge whether the two stimuli of each pair were identical or not. A total of 40 stimuli were presented in a random order.

#### 3.7.2. Results

In this task, our patient scored 100% correct.

### 3.8. Perceptual matching of complex figures

#### 3.8.1. Material and methods

We used the same "daisies" employed in the third constructional task. In this case two stimuli were aligned one above the other: in half trials the two stimuli were identical, and in half trials the stimuli differed for one element (one daisy lacked one petal). The patient was required to judge whether the two stimuli were identical. A total of 84 trials were given in a random order.

#### 3.8.2. Results

In this task, our patient scored 76/84 correct (90%). G.N. made 7 errors with different stimuli, but error distribution did not show a specific relation with spatial orientation of missing elements.

### 3.9. Delayed matching of complex figures

#### 3.9.1. Material and methods

The same stimuli employed above were used in a delayed matching task. In this case only one stimulus was shown for 10 seconds and then, after a 10-sec delay the patient was presented with a second daisy. The task consisted in judging whether the two stimuli of each pair were identical or not. A total of 84 trials were given in a random order.

#### 3.9.2. Results

In this task, our patient responded correctly for 73/84 items (87%). Most errors (9/11) were made with different stimuli, but did not show any systematic spatial orientation.

### 3.10. Perceptual-imaginative task

#### 3.10.1. Material and methods

Only one daisy was presented at a time; in half trials one petal was missing, whereas in half trials entire stimuli were presented. The patient was required to judge whether each stimulus was complete or whether any petal was missing; this were the case the patient was asked to point to the location of the missing petal. A total of 48 stimuli were presented in a random order.

#### 3.10.2. Results

The patient did not show difficulties in identifying incomplete daisies and in pointing to missing petals, irrespective of their location.

## 4. Discussion

The patient described here was affected by a selective difficulty in producing complex figures when he had to draw horizontal elements, independently from number, shape and spatial position of stimuli. The special neuropsychological assessment revealed that neither tracing single lines in whatever direction, nor visuoperceptual performance were affected by any relevant impairment. Therefore, the difficulty in drawing horizontal elements embedded in complex patterns seemed to be selectively related to a dissociation between the construction of horizontal lines (impaired) and the production of oblique or vertical lines (spared). Analogously, a difficulty in planning constructional movements along horizontal dimension might explain the poor performance on the preliminary "draught-board task", that was instead performed successfully by following oblique coordinates.

It would be possible to question whether an object-centered neglect [8] could explain the frequent omissions of left-sided horizontal elements in copying "daisies" and "windmills", and even the difficulty in switching between boxes to construct a draught-board following horizontal or vertical coordinates. However, the patient described here did not show relevant asymmetries in visuoperceptual or representational tasks. Therefore, one should ascribe the patient's errors to a specific object-centered neglect that manifested only when the patient was involved in constructional tasks (see [9]). This hypothesis would require several post-hoc assumptions, and it would hardly explain the constructional errors in which the patient reproduced all the stimulus elements, but in wrong spatial orientation (e.g., the tendency to reproduce horizontal lines as oblique in copying asterisks).

The data reported here seem to be better consistent with the hypothesis that our patient showed a specific impairment in processes involved in drawing. The difficulty in drawing horizontal elements of complex figures might be ascribed to defective use of a reference frame involved in reproducing spatial relationships among single components of multipart figures. In this context, the present data would demonstrate that the horizontal coordinate system of reference might be impaired independently of oblique and vertical coordinate systems, in spite of the fact that computation of oblique orientation is often considered more difficult than processing of horizontal or vertical dimensions [5, 6]. Normal human subjects are better at detecting, discriminating and remembering horizontal and vertical stimuli than those that with oblique orientation (for reviews, see [10,11]). In the visual modality, such an "oblique effect" seems to arise at a level higher in the visual system than the retina [12], and would suggest that oblique orientations have a more complex representational structure than cardinal orientations do [5, 11,13,14]. The dissociation of spatial representation along horizontal, vertical, and radial dimensions, as that reported here, is more consistent with cognitive models according to which the mental representation along each of the three axes is derived from the integration of different modality dependent information [15].

In conclusion, it is possible to suggest that the oblique coordinate system of reference operates independently of vertical and horizontal coordinate systems in building a complex figure and that, therefore, these two systems do not constitute a reference norm to define oblique orientation, as previously suggested [16]. The possible neural underpinnings of processing of the oblique dimension might be functionally and anatomically separate from those representing the vertical and horizontal axes, but such speculation warrants specific neurofunctional studies.
